# Preliminary Results of a Novel Standardized Technique of Femtosecond Laser-Assisted Deep Anterior Lamellar Keratoplasty for Keratoconus

**DOI:** 10.1155/2020/5496162

**Published:** 2020-09-03

**Authors:** Andrea Lucisano, Giuseppe Giannaccare, Marco Pellegrini, Federico Bernabei, Angeli Christy Yu, Adriano Carnevali, Laura Logozzo, Giovanna Carnovale Scalzo, Vincenzo Scorcia

**Affiliations:** ^1^Department of Ophthalmology, University Magna Graecia of Catanzaro, Viale Europa, 88100 Catanzaro, Italy; ^2^Ophthalmology Unit, S. Orsola-Malpighi University Hospital, Via Palagi 9, 40138 Bologna, Italy; ^3^University of Ferrara, Department of Morphology, Surgery and Experimental Medicine, 44121 Ferrara, Italy

## Abstract

**Purpose:**

To evaluate the feasibility and the initial outcomes of a novel standardized surgical technique of femtosecond laser- (FSL-) assisted big-bubble deep anterior lamellar keratoplasty (BBDALK) for eyes with keratoconus.

**Methods:**

This prospective interventional case series included 11 consecutive FSL-assisted BBDALK procedures performed for the eyes with keratoconus from September 2019 to December 2019. The FSL was used to create (i) an intrastromal channel incision (1.7 mm in length, 4.6 mm in width, 80% depth, and cut energy of 1.70 *μ*J) and (ii) a 9.0 mm diameter circular lamellar side cut 65 *μ*m above the endothelium (cut energy of 0.90 *μ*J) intersecting the intrastromal incision. In the operating room, a blunt dissector was used to open the intrastromal channel incision, through which a blunt spatula was inserted, tangentially advanced towards the center of the cornea, and replaced with a blunt cannula for pneumatic dissection. The subsequent surgical steps did not differ from the conventional technique. Main outcome measures were the success rate of pneumatic dissection and the percentage of intraoperative complications.

**Results:**

Eleven eyes of 11 patients (6 males and 5 females; mean age: 34.54 ± 13.23 years) underwent FSL-assisted DALK. Using the FSL, both corneal incisions (lamellar side cut and intrastromal channel incision) were successfully created in all cases without the need for repeat docking or additional dissection. Pneumatic dissection with type 1 bubble formation succeeded in all 11 eyes (100%). DALK surgery was completed uneventfully in all cases. Descemet membrane perforation did not occur in any case, and no procedure was converted to penetrating keratoplasty.

**Conclusion:**

Using standardized FSL parameters for both incision design and cut energy in BBDALK surgery, pneumatic dissection can be achieved in a very high rate of cases with minimal risk of intraoperative complications.

## 1. Introduction

Deep anterior lamellar keratoplasty (DALK) has been recognized as the first-line surgical procedure for eyes with corneal stromal disease but healthy endothelium [1, 2]. The main advantages of DALK over penetrating keratoplasty (PK) include the elimination of endothelial rejection, reduced endothelial cell loss, and improved long-term graft survival [3–8]. Furthermore, since DALK is essentially an extraocular procedure, the complications associated with an open-sky surgery are avoided [1, 8, 9].

However, based on the 2019 statistical report of the Eye Bank Association of America [10], the general popularity of DALK has remained limited with only 11% of corneal transplants for keratoconus performed using DALK compared to 89% with PK. Though several techniques for DALK have been proposed [1], there has been a slow adoption among corneal surgeons, mainly due to technical challenges in achieving a smooth and regular graft-host interface compatible with optimal vision [11].

Currently, one of the most commonly used methods is the big-bubble (BB) technique, which involves intrastromal injection of air to obtain a cleavage plane between the deep stroma and either the pre-Descemetic layer (PDL) through a type 1 bubble or Descemet's membrane (DM) through a type 2 bubble [12]. Although successful pneumatic dissection results in favourable visual and refractive outcomes, the success rate of pneumatic dissection is variable even in the hands of experienced surgeons ranging from 64 to 91%, depending on the technique employed and/or the type and severity of underlying corneal disease [13–18].

Using intraoperative optical coherence tomography (OCT) imaging, we have previously demonstrated that what is critical for successful BB formation is the depth at which pneumatic dissection is attempted. When the cannula reaches within 100 *μ*m of the posterior corneal surface, the likelihood of successful big-bubble formation exceeds 90% [19]. Based on this surgical principle, the femtosecond laser (FSL) has been proposed to make intracorneal incisions of appropriate depth, which in turn serve as a guide for cannular insertion and subsequent pneumatic dissection. In this pilot study, we evaluate the feasibility and initial outcomes of the first series of keratoconus eyes operated on with this novel standardized technique of FSL-assisted BB-DALK.

## 2. Materials and Methods

This prospective interventional case series evaluated the outcomes of consecutive FSL-assisted BBDALK procedures performed in eyes with keratoconus from September 2019 to December 2019 at a single tertiary referral center (Department of Ophthalmology, University of Magna Graecia, Catanzaro, Italy). The study adhered the tenets of the 2013 Declaration of Helsinki and was approved by the local ethics committee (Comitato Etico Regione Calabria—Sezione Area Centro). Written informed consent for the surgery and research was obtained from all participants.

All cases required corneal transplantation for unsatisfactory corrected distance visual acuity (CDVA) due to significant refractive errors and/or poor tolerance to rigid gas permeable contact lenses. Eyes with previous hydrops, evident lesions at the level of DM and endothelium, and history of trauma or other ocular diseases were excluded. Preoperatively, all patients underwent a complete ophthalmologic evaluation including CDVA testing, slit-lamp examination, and anterior segment optical coherence tomography (AS-OCT, Casia; Tomey, Tokyo, Japan). CDVA was recorded using the Snellen visual acuity chart. All operated patients were evaluated 6 months after surgery. The main outcomes were the success rate of pneumatic dissection as well as the percentage of intraoperative complications. Secondary outcomes were postoperative complications, final CDVA, and refractive results.

### 2.1. Surgical Technique

**Figure 1:**
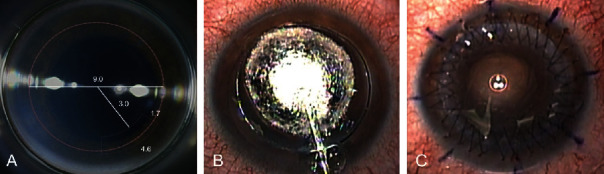
Intraoperative and postoperative images of a representative patient who underwent femtosecond laser- (FSL-) assisted big-bubble deep anterior lamellar keratoplasty: (a) shape and size of FSL incisions; (b) successful formation of big bubble; (c) end of the surgery with keratoscopy control of final astigmatism.

FSL-assisted BB-DALK surgery was performed in all eyes by a single high-volume corneal surgeon (V. S.) as demonstrated in Video 1 (Supplemental Digital Content). In all cases, anaesthesia and akinesia were obtained by means of peribulbar injection of 10 mL of a 0.75% ropivacaine solution. A single drop of tropicamide 1% (Visumidriatic 1%, Visufarma, Roma, Italy) was instilled preoperatively to induce pharmacologic mydriasis and improve intraoperative visualization [20]. All laser treatments were performed using the Victus FSL platform (Bausch & Lomb, Bridgewater, NJ, USA). The FSL parameters of the corneal incisions were calibrated based on the corneal thickness in the area of intended cuts using the real-time swept-source OCT imaging integrated into the FSL platform. Applying the software developed and approved for intracorneal ring segment (ICRS) implantation, the intrastromal channel incision parameters were set to 1.7 mm in length, 4.6 mm in width, and 80% depth at the superior cornea, usually at 10-11o'clock position, using 1.70 *μ*J of cutting energy. The inner edge of this channel was 3.0 mm from the center of the cornea. Using 0.90 *μ*J of cutting energy, the 9.0 mm diameter circular lamellar side cut was designed to intersect with the first planar incision, leaving a residual thickness of 65 *μ*m above the endothelium (Figure 1(a)).

In the operating room, a blunt dissector (Model JDBB01, E. Janach, Como, Italy) was used to open the intrastromal channel incision. The blunt spatula (Model AE-2900, Asico, Westmont, USA) was inserted through the intrastromal channel and advanced tangentially to the cornea posterior surface towards the center of the cornea, maintaining the same depth of the entrance plane. The spatula was then replaced with a blunt 27-gauge Fontana cannula (Model J2641.58, E. Janach, Como, Italy), and pneumatic dissection was attempted (Figure 1(b)). Following debulking of about 80% of the anterior stroma, the roof of the bubble was incised using a 30° blade under viscoelastic (IAL-F, Fidia, Padova, Italy) protection. The slit of the incised bubble was enlarged through blunt Vannas scissors, and removal of the bubble roof was completed using corneal scissors. The donor cornea was punched from the endothelial side with a Barron donor punch (Katena Products, Inc., Parsippany, NJ, USA) to the same diameter as the recipient cornea (9.0 mm). After staining with 0.06% trypan blue dye (VisionBlue; D.O.R.C., Zuidland, the Netherlands), DM and endothelium were gently stripped off using a dry Weck-Cel sponge. Four interrupted 10-0 nylon sutures initially secured the graft into the recipient bed, and the graft was sutured into the recipient bed with 16-bite double running, 10-0 nylon suture. The astigmatism was checked under the guidance of a microscope-mounted digital keratoscope (Figure 1(c)).

Starting the following day, betamethasone 0.2% and chloramphenicol 0.5% eye drops were administered every 2 hours for 1 week. Subsequently, antibiotic treatment was discontinued while dexamethasone 1 mg/ml was prescribed 4 times daily and then slowly tapered off during the following 6 months.

### 2.2. Data Analysis

Statistical analysis was performed using SPSS Statistics (SPSS, Inc., Chicago, IL) for data analysis. Values were expressed as mean ± standard deviation (SD). The Wilcoxon signed-rank test was used to compare the continuous variables. A *p* value less than 0.05 was considered statistically significant.

## 3. Results

This series included 11 eyes of 11 patients that underwent FSL-assisted DALK. The mean age at the time of surgery was 34.54 ± 13.23 years, and 6 patients (55%) were males. Based on the Amsler–Krumeich classification, 4 cases (36%) were classified as stage II, 5 (45%) as stage III, and 2 (18%) as stage IV. All cases had a follow-up of at least 6 months (7.2 ± 1.5 months).

Utilizing the FSL, both corneal incisions (lamellar side cut and intrastromal channel) were successfully created in all cases without the need for repeat docking or additional dissection. Pneumatic dissection with type 1 bubble formation succeeded in all 11 eyes (100%). DALK was completed uneventfully in all cases. DM perforation did not occur in any case, and no procedure was converted to PK.

Preoperative mean Snellen CDVA significantly increased from 0.34 ± 0.11 to 0.58 ± 0.07 at final follow-up (*p* < 0.001), while mean keratometric astigmatism and mean *K* steep significantly decreased from 3.71 ± 1.95 to 2.40 ± 0.57 diopters (*D*) (*p*=0.04) and from 55.20 ± 3.09 to 45.60 ± 1.35 *D* (*p* < 0.001), respectively. Complete attachment of the donor lamella was achieved with corneal clarity restored in all cases. No episode of double anterior chamber formation, immunologic rejection, graft failure, or any other postoperative complications was observed.

## 4. Discussion

Although DALK has clear advantages over PK in terms of graft survival, technical challenges of the procedure along with poor reproducibility still limit its widespread adoption among corneal surgeons [21, 22]. Injecting air at the proper depth using a reproducible technique represents the significant surgical challenge, especially among novice corneal surgeons [23].

Though several studies have described the use of FSL for the creation of an intrastromal channel for the air injection [24–27], FSL settings for DALK have not been standardized thus far. Unlike FSL-based procedures for cataract surgery and ICRS, no dedicated software has been developed, possibly due to technical challenges and concerns regarding the safety and efficacy of FSL for creating DALK incisions. In fact, laboratory studies have demonstrated that FSL creates uneven interfaces at greater corneal depths (as required during DALK surgery) and may possibly induce endothelial cell damage [28–30].

In this study, we have demonstrated the feasibility of FSL-assisted BB-DALK according to our standardized technique. Firstly, the FSL has allowed to create precise and large-diameter side cut incision. At the 9 mm optical zone, the cornea tends to be less affected by ectasia and more regular with less variability in zonal pachymetry. Consequently, a deeper incision based on the thinnest point pachymetry is created and could account for the consistent surgical outcomes observed. Secondly, the FSL has allowed the creation of accurate and reproducible intrastromal incisions that represents a deep entrance plane for the advancement of the cannula at the appropriate depth, thereby resulting in high rates of successful big-bubble formation in this series. Unlike conventional DALK using manually calibrated trephines, FSL allows a precise deep trephination, which is associated with high rates of successful pneumatic dissection independent of surgical experience [18], eliminating the risk of perforation in this surgical step. Interestingly, pneumatic dissection resulted in the formation of a type 1 bubble in all cases. The presence of PDL confers additional strength to the floor of the bubble and accounts for the absence of intraoperative and postoperative complications observed in this series despite the inclusion of eyes with more advanced stages of keratoconus [11, 31]. Although the FSL settings used were originally developed for ICRS implantation, the modifications presented in this series could represent a major improvement in the DALK technique.

Another important feature of this procedure is the design of the intrastromal channel. Instead of a straight narrow tunnel, we created a large intrastromal pocket that is more easily identifiable by the surgeon in the operating room. Additionally, a large stromal pocket would allow insertion of the cannula through a different intrastromal tunnel in case of failure of the first attempt. In order to prevent air from escaping through the large intrastromal channel, before injecting air for bubble formation, the cannula was advanced tangential to the corneal posterior surface maintaining the same depth of the entrance plane. Though limited follow-up is currently available, the significant improvements of CDVA and keratometric outcomes in this series seem to support other studies that have reported stable and faster wound healing after FSL-assisted DALK [32, 33]. However, although the visual and refractive outcomes of FSL-assisted DALK have been reported as comparable to conventional DALK, further studies are required to evaluate long-term differences.

Besides FSL, other approaches have been described to assist surgeons during BB-DALK and improve the success of the pneumatic dissection. For instance, ultrasound pachymetry and intraoperative OCT are helpful tools to achieve a proper depth of air injection [34–36]. However, the former approach appears less precise and standardized compared to our technique and requires dedicated instruments such as a corneal pachymeter and a micrometer-controlled knife [34]. The latter approach is useful in cases with decreased visualization under the operating microscope, but the main limitation is represented by the obstruction of the OCT image acquisition by metallic instruments [35]. As demonstrated by our preliminary results, FSL may help to overcome these limitations, thereby improving the pneumatic dissection success rate while maintaining high standards of safety. In addition, this technology may increase the safety and reproducibility of the circular lamellar trephination.

Although the present case series is an appealing attempt at standardizing FSL-assisted BB-DALK, there are some limitations in this study. Firstly, this is a pilot study with limited sample size and no control group. Larger studies are required to validate these data, and randomized clinical trials are desirable to establish the superiority of this approach over the conventional DALK. Moreover, long-term longitudinal observation is required to evaluate the influence of FSL-assisted DALK on postoperative refractive and visual outcomes. Though the laser setting used in this series was developed for ICRS implantation, the promising initial surgical outcomes of FSL-assisted DALK support the need for the development of dedicated settings for DALK. With greater understanding of the principles behind successful BB formation [19], FSL settings can be further optimized by improving the reproducibility and overall outcomes of the procedure.

In conclusion, using standardized FSL parameters for both incision design and cut energy in DALK surgery, pneumatic dissection can be achieved in a very high rate of cases with minimal risk of intraoperative complications.

## Data Availability

The data used to support the findings of this study are included within the article.

## Supplementary Materials

Supplementary Materials Channel incision parameters are set to 1.7 mm in length, 4.6 mm in width, and 80% depth at the superior cornea. A 9.0 mm diameter circular lamellar side cut is designed. In the operating room, a blunt spatula is inserted, then it is replaced with a blunt 27-gauge cannula, and pneumatic dissection is attempted. Subsequently, the bubble roof is removed. In the donor cornea, the Descemet membrane and endothelium are stripped off after staining with trypan blue dye. Finally, the graft is sutured into the recipient bed with 16-bite double running sutures. The astigmatism is checked under the guidance of a microscope-mounted keratoscope.
